# Opinion: more mouse models and more translation needed for ALS

**DOI:** 10.1186/s13024-023-00619-2

**Published:** 2023-05-04

**Authors:** Elizabeth M.C. Fisher, Linda Greensmith, Andrea Malaspina, Pietro Fratta, Michael G. Hanna, Giampietro Schiavo, Adrian M. Isaacs, Richard W. Orrell, Thomas J. Cunningham, Abraham Acevedo Arozena

**Affiliations:** 1 UCL Queen Square Motor Neuron Disease Centre, UCL Queen Square Institute of Neurology, University College London, Queen Square, London, WC1N 3BG UK; 2 Department of Neuromuscular Diseases, UCL Queen Square Institute of Neurology, University College London, Queen Square, London, WC1N 3BG UK; 3 UK Dementia Research Institute at UCL, London, WC1E 6BT UK; 4 Department of Neurodegenerative Disease, UCL Queen Square Institute of Neurology, University College London, Queen Square, London, WC1N 3BG UK; 5 Department of Clinical and Movement Neurosciences, UCL Queen Square Institute of Neurology, University College London, Queen Square, London, WC1N 3BG UK; 6 MRC Prion Unit at UCL, Courtauld Building, 33 Cleveland Street, London, W1W 7FF UK; 7 Research Unit, Hospital Universitario de Canarias, ITB-ULL and CIBERNED, La Laguna, 38320 Spain

**Keywords:** Amyotrophic lateral sclerosis, Motor neuron disease, Mouse models, Patient stratification, Translation

## Abstract

Amyotrophic lateral sclerosis is a complex disorder most of which is ‘sporadic’ of unknown origin but approximately 10% is familial, arising from single mutations in any of more than 30 genes. Thus, there are more than 30 familial ALS subtypes, with different, often unknown, molecular pathologies leading to a complex constellation of clinical phenotypes. We have mouse models for many genetic forms of the disorder, but these do not, on their own, necessarily show us the key pathological pathways at work in human patients. To date, we have no models for the 90% of ALS that is ‘sporadic’. Potential therapies have been developed mainly using a limited set of mouse models, and through lack of alternatives, in the past these have been tested on patients regardless of aetiology. Cancer researchers have undertaken therapy development with similar challenges; they have responded by producing complex mouse models that have transformed understanding of pathological processes, and they have implemented patient stratification in multi-centre trials, leading to the effective translation of basic research findings to the clinic. ALS researchers have successfully adopted this combined approach, and now to increase our understanding of key disease pathologies, and our rate of progress for moving from mouse models to mechanism to ALS therapies we need more, innovative, complex mouse models to address specific questions.

## ALS is complicated -- partly because it is not one disease

Amyotrophic lateral sclerosis (ALS) is a devastating, progressive, incurable neurodegenerative disorder that typically strikes in mid-life [1, 2]. Patients may present initially with spinal or bulbar-onset and studies in Europe show more spinal-onset in men and more bulbar onset in women, with bulbar-onset being overall lower in Asia than Europe [3]. ALS is slightly more common in men than women, with an estimated lifetime risk in UK and USA of ~ 1 in 350 for men and 1 in 450–500 for women [4]. Death, usually from respiratory failure, typically occurs within 3–4 years of diagnosis; however, this timing and many other features including incidence are population-specific, for example, ALS may be less common in people with African ancestry [5, 6]. Motor neurons are the primarily affected cells in ALS, but the disease lies on a spectrum with frontotemporal dementia, and clearly other cell types are involved. Semantic dementia and cognitive and/or behavioural changes may affect up to 50% of ALS patients [3]. Disease heterogeneity manifests in the clinic with the common observation of patients reaching end-stage disease from symptom onset within one year, and other patients progressing slowly over more than a decade. These phenotypic extremes of ALS are likely to be modulated by different mechanisms and/or by different rates of pathological changes and motor cell demise [7].

In nearly all cases of ALS, motor neurons carry cytoplasmic inclusions of the protein TDP-43 (‘TDP-ALS’). This occurs regardless of whether the disease is ‘sporadic’ ALS, arising from no known cause, or is driven by monogenic mutations in the ~ 30 known ‘ALS genes’ (familial ALS), which include *TARDBP*, the gene encoding TDP-43 that is mutated in < 5% familial ALS. Exceptions are patients carrying monogenic mutations in *SOD1* and *FUS*, in whom SOD1 and FUS proteins are found, respectively, in pathogenic inclusions. At least 40 genes associated with ALS, including those likely conferring increased risk, have been described [1]. ALS mutations are usually, but not always, dominant, but may have variable penetrance, indicating modulation by genetic background and/or unknown environmental factors [2, 3, 8]. Thus, while the clinical and cellular outcomes of sporadic or familial ALS maybe similar, the upstream causes may be different, and this remains a key issue for translation.

Most of our mechanistic understanding of ALS comes from investigating cases that have a clearly defined genetic cause, but we have no unified picture of pathomechanism because the proteins encoded by ALS genes have diverse functions. For example, RNA metabolism is disrupted by mutations in RNA binding proteins, including TDP-43, FUS, HNRPNA1, MATR3, SETX; whereas deregulation of autophagy/protein degradation and membrane trafficking may be the primary mechanism arising from mutations in OPTN, TBK1, SQSTM1 and VCP [3], noting that multiple mechanisms likely contribute to motor neuron death. Three treatments for ALS, riluzole, edaravone and AMX0035 are approved in different geographical areas but these drugs only prolong life by a few months in some patients and may not be appropriate for all individuals [3]. New antisense oligonucleotide (ASO) treatments offer hope for patients with genetic forms of ALS, including *SOD1*-ALS, *FUS*-ALS and potentially *C9orf72* expanded hexanucleotide repeat-ALS, which constitutes up to 40% of familial cases [9–13]. In addition, the genes *STMN2* (*STATHMIN* 2) and *UNC13A*, which have altered splicing patterns in TDP-ALS, are now exciting potential targets for genetic therapy in *sporadic* ALS [3, 10, 14–17].

Therapeutic strategies, including gene modifying treatments, may target only one of several different biological pathways involved in disease processes within each ALS subtype. These pathways can be non-neuronal – for example, one approach to new therapeutics is focussed on the emerging role of the immune response in neurodegenerative processes and possible effects on rate of neuronal loss. In ALS a reduction of T regulatory cell numbers is linked to a faster progression of the disease along with an increased intrathecal T-cell activation [18]. T regulatory cell function helps maintain immune homeostasis and self-tolerance, and recently two independent reports showed that immune dysregulation linked to lymphocyte function in ALS may be driven by cell senescence [19, 20]; elevated senescent lymphocytes in the circulation are found in ALS animal models and ALS patients (ALS4, arising from senataxin mutations) [19, 20]. Another example of a novel non-neuronal therapeutic target -- with aligned human and mouse data -- is the gut bacteriome, which modulates the immune response [21]; bacteria present in the human gut can improve survival of an ALS mouse model [22].

ALS is therefore a diverse disorder with variable presentation, genetics, pathogenesis, histopathology and family history. Currently, we have limited insight into the environmental and/or genetic factors likely to be involved in sporadic ALS [2]. While remarkable progress has been made in identifying a wide range of pathological and molecular changes in human subjects, ex vivo, in vitro and animal models, there is little consensus on which mechanisms are *key* in the heterogeneous subtypes of ALS that result in motor neuron death -- which are common and which are specific to different subtypes, and which are important for early versus late disease stages. This reflects the situation found in earlier days of cancer research, which started with models obtained by mutating causative genes, but then progressed to tailoring complex combinations of engineered alleles in mouse to help dissect individual pathways and pathomechanisms in different cancer subtypes.

## There is no one-size-fits-all for mouse models of ALS

The first ALS gene discovered was *SOD1* in 1993 [23], and this seminal research finding was followed up in 1994 by creation of the first ALS mouse model, a mutant *SOD1* transgenic [24]. The next major effect ALS genes, *TARDBP* and *FUS* were not published until over ten years later [25, 26]. As a result, for over a decade *SOD1* overexpressing transgenic animals were the only available models of ALS and therefore, due to the need for treatments, were extensively used for therapeutics development by academia and industry.

Transgenic *SOD1* mutant overexpressing mouse strains generally develop a rapid disease and are a faithful ALS model, largely mimicking the course of the human disorder. However, *SOD1*-ALS accounts for only < 2% of human ALS, and now appears to be a possible pathomechanistic outlier in the complex ALS landscape, because *SOD1*-ALS tissue does not show the TDP-43 deposition seen in most ALS cases; for the same reason *FUS*-ALS may also be an outlier. Thus, ALS therapies that have been developed and tested in SOD1 transgenics, may not be applicable to patients with different ALS subtypes. However, it remains imperative to study SOD1-ALS models because they recapitulate the progressive loss of motor neurons that define all ALS cases, and so may be useful for identifying possible common pathological pathways in different forms of ALS, and of course they are essential for addressing the 20% of familial cases with this subtype.

*TARDBP*-ALS and *FUS*-ALS proved more difficult to model because these genes are dosage-sensitive (i.e., more, or less, than two copies of these genes in the genome will cause an aberrant phenotype), and transgenic mice usually overexpress their transgene because of the method used to make the animals [27, 28]. For example, even low-level overexpression of *wild-type* TDP-43 within a transgenic mouse results in late onset neuromuscular junction damage and hindlimb paralysis [29]. Furthermore, the endogenous mouse loci remain intact, which means loss of function effects cannot be modelled in conventional transgenic mice unless they are bred to knockout animals [27].

A better strategy to engineer *TARDBP*-ALS and *FUS*-ALS models may be to generate animals expressing a normal ‘physiological’ dose from a single copy of the gene. Several mouse strains bearing mutations in endogenous ALS genes created by random mutagenesis or targeted (knocked in) approaches into the mouse gene are currently available [27]. For example, of the *Tardbp* endogenous models, the M323K mutation that arose from chemical mutagenesis shows, when homozygous, progressive mild motor neuron death and gain of function splicing changes [30]. Of the gene-targeted mutations into *Tardbp*: a Q331K mutation [31] has cognitive dysfunction and gives new insight into TDP-43 autoregulation, and potentially into the link between ALS and FTD [32]; homozygous M337V and G298S mutations affect neuromuscular junctions and produce spinal cord gliosis at late stages, without frank neurodegeneration [33]; an N390D mutation is reported to have a full spectrum of TDP-43 pathology including TDP-43 aggregation and motor neuron degeneration in heterozygotes [34].

These knock-in mouse models solve the issue of dosage sensitivity, but do not tackle the different regulation/isoforms that may occur in human and mouse genes. New ‘genomically humanised’ knock-in models -- in which the mouse locus is replaced with the human orthologous sequence -- can include exons and introns of the corresponding ALS gene – for example, as in current models for *FUS*-ALS [10, 35–37]. Such genomic humanisation usually results in the human, rather than mouse, splice isoforms, noting that human genes typically have more complex splicing patterns than their mouse orthologues [38, 39].

Humanised and mouse knock-in strains may provide new insights because they are physiological models that can show us the early stages of ALS before overt disease manifests. For example, slow FUS knock-in models appear representative, at least of *FUS*-ALS early changes [10, 35–37]. In contrast, while the SOD1 transgenics have been workhorses in the field of ALS research and necessary for development of current potential therapies including anti-SOD1 ASOs, they usually have a fast disease course and are not necessarily an appropriate model for studying pre-symptomatic stages of disease [40]. Furthermore, testing therapeutics targeting the pathogenic gene product in an overexpression model may not provide helpful data for modulating physiological levels of the same biological target [41].

However, in physiological models motor neuron degeneration often starts in mid-life for a mouse, at ~ 12-months of age, and leads to progressive but mild phenotypes. Although it would be interesting to ascertain the determinants of this mid-life onset, these models are slow and therefore costly to use for drug development, and from our experience, they are not adopted by pharma/biotech and are widely regarded as ‘too subtle’ for use in drug discovery [41]. Furthermore, studying such mice within the typical 3- or 4-year tenure of a postdoc or European PhD student project can be challenging given the need for publication. Thus, there is a tension between the accuracy of the model versus the constraints of academia and pharma/biotech, part of which may be effectively addressed by measuring reliable endophenotypes and novel biomarkers rather than time to death. Of course, this situation is not unique to ALS research, but can result in new potential drugs being tested in models to fit time and financial constraints rather than the biology of human disease.

Perhaps one of the biggest current challenges for ALS research is that while we can generate mouse models of familial disease, much of the fundamental biology of ALS remains unclear and many different cellular pathways have been mechanistically implicated in pathology. Without knowing the *key* pathological processes, it difficult to create animal models reflecting critical ALS pathophysiology to support drug development [42, 43].

In addition, mouse models are based on genetic forms of ALS and therefore do not address the majority of human disease that is sporadic. However, most sporadic ALS cases show TDP-43 loss of nuclear function – likely leading to common pathological outcomes, including splicing dysregulation, which results in the inclusion of cryptic exons in many transcripts, such as those of the *STMN2* or *UNC13A* genes [14–17, 44]. These exons may not exist in the mouse but the human sequences can be knocked into the mouse genome, and experimental data show this can give rise to the same splicing dysfunction – inclusion of cryptic exons - in ‘genomically humanised’ mouse models [14–17, 44]. Thus, it is now possible that important human molecular pathology of most familial and sporadic ALS subtypes may be modelled in mice, so opening the door for translation of genetic and other therapies based on modulating genes affected by TDP-43 loss of function.

As protective loci are identified, including in non-coding DNA, these too may be modelled potentially paving the way for new therapies to ameliorate disease [45]. In these experimental/translational platforms, we note the need for essential control animals – for example, those that are humanised with the normal, wild-type, human sequence, without mutations and on isogenic backgrounds, to determine if the human sequence per se produces an aberrant phenotype in the mouse models.

Experimentalists facing the difficult choice of which is the most suitable ALS mouse model for their research may take advantage of available information, such as the comprehensive list of mouse models published by us in 2019 [27], or a more recent review that includes new mouse and rat models by Todd and Petrucelli [28]. Additionally, some key models and their main phenotypic features are listed on the Alzforum ALS model site (www.alzforum.org/research-models/als). All models can only ever address aspects of the human disease and provide a snapshot on a particular genetic background within a specific environment. Furthermore, each model should be used only if it is the best fit for answering a biological question – *thus there are many more, useful models to be created for ALS research.*

## The lack of TDP-43 deposition in Tardbp/TARDBP mouse models

Pathological cytoplasmic inclusions of FUS, TDP-43 and SOD1 characterise different human ALS subtypes. However, FUS and TDP-43 mutant mice develop progressive motor neuron loss generally without showing cytoplasmic accumulation of these proteins, underlining that these inclusions are not a necessary step for the initiation of neuronal loss, although they might be critical for late-stage disease -- TDP-43 deposition may be a marker of end-stage disease and may not contribute to early pathogenesis, although early loss of TDP-43 nuclear function is likely critical for neurodegeneration. However, we note an older transgenic model is reported to develop TDP-43 aggregation without neurodegeneration [46].

Crucially, TDP-43 deposition is not specific for ALS, as it has been found in other disorders such as Alzheimer’s disease [47], polyglutamine diseases [48], limbic-predominant age-related TDP-43 encephalopathy (LATE) [49], and as a result of neurological insults, including methyl mercury exposure [50] and hypoperfusion [51]. Also, TDP-43 in human tissue is subject to post-mortem delay, and a recent report notes that aggregates in human ALS and FTD samples are different from the ‘anisosomes’ seen in freshly processed mouse tissues [52]. Nevertheless, while mutant *Tardbp* can cause disease without aggregation [30, 31, 53], being able to recapitulate TDP-43 deposition in mice would be of great use for modelling the full gamut of ALS pathology, as reported in N390D KI mice [34]. Approaches for modifying ALS mouse models to produce TDP-43 deposition are being explored, including injection of TDP-43 seeding aggregates into mice expressing human TDP-43 [54] to develop potential immunotherapy [55], or genetic strategies to mis-localize TDP-43 to the cytoplasm, for example, by mutating its nuclear localization signal [56, 57].

With respect to the use of mouse models for ALS research, the issue of TDP-43 deposition is a good example in which complex models with more than one mutation may be required to investigate human pathological processes.

## We do not expect exact face validity from ALS mouse models

The value of animal models lies in defining the key evolutionarily conserved biochemical pathways that maintain normal function but which are aberrant in disease. However, clearly each species is different and we should not expect gross ‘face validity’ from mouse models: the physiology, lifespan, size, and genetic background of a mouse will impact how a disease mutation manifests. We make mouse models to understand molecular pathways and cellular outputs arising in disease, not to exactly mimic the human condition.

Factors affecting mouse models of ALS include the significant anatomical differences in the motor system of rodents and primates, for example, in the corticospinal tract (CST). The CST is a white matter motor pathway running from the cerebral cortex to the spinal cord which is responsible for voluntary movements of the limbs and trunk, including control of skilled voluntary movements. In primates, the CST descends in the ventral and lateral segments of the spinal cord, and forms monosynaptic connections with lower motor neurons (LMN) in the ipsilateral anterior grey horn. Lower motor neuron axons leave the spinal cord through the ventral root to form peripheral nerves, which innervate the musculature of the body. In contrast, in rodents the CST descends only in the dorsal spinal cord, and unlike in primates, the CST fibres do not form monosynaptic connections with spinal motor neurons; there are therefore no direct cortico-motor neuronal connections in rodents. This anatomical difference between mice and humans has implications for modelling ALS in mice as the diagnosis of ALS is based on the presence of both upper motor neuron (UMN) and LMN signs, and we cannot exactly model UMN degeneration of the CST.

Another obvious example of mouse:human difference is that the humane endpoint for mouse studies does not equate to endstage disease in humans [57]. Thus, with respect to the use of mouse models of ALS, we may not see exact correspondence to human timing, neuroanatomy and physiology, nevertheless, the great value of these models is the unparalleled insight they give us into molecular pathways in vivo and other potential therapeutic targets including causative genes themselves.

## Working with complex mouse models – lessons from cancer research

Creating novel, complex models tailored to address specific questions helps us re-create human pathomechanisms and focus on the most severely affected pathways and events. Using complex models has become standard in cancer research since this strategy can offer major advances in understanding pathology and provide unexpected novel insights.

For example, in considering approaches to studying a single major disease gene, the p53 tumour suppressor protein (encoded by the *TRP53* gene) is mutated in ~ 50% human cancers, and the remaining 50% usually carry mutations that inactivate the p53 pathway; thus, there has been interest in reactivating p53 as a treatment for cancer [58]. However, this requires detailed understanding of tumorigenic pathways and its timing, as well as the genetic mechanism enabling cells to ‘switch on’ a normal copy of p53 in vivo within the cancer environment. In a series of outstanding experiments using mice with carefully designed mutations that include those giving temporal control of p53 expression in established autochthonous tumours, researchers have used immunocompetent mouse models bearing complex mutations to show that spontaneous tumours arising in the context of p53 deficiency were extremely sensitive to reactivation of p53 [58–60]. Critically, these effects turned out to be stage-specific: in lung tumours, p53 reactivation eliminates only highly advanced lesions, sparing those of low-grade [58–60]. Mutant strains created for these studies were engineered with oestrogen receptor-regulatable systems, inducible-reversible RNAi paradigms, and floxed conditional mutations, which allowed researchers to show that p53 mediates different tumour-suppressive responses in different cancer types at different stages of progression, ultimately demonstrating the potential of p53 reactivation in the battle against cancer [58, 61, 62].

Another study, focussed on colorectal cancer, investigated the competitiveness of wild-type versus mutant cells, a theme with relevance to the non-cell autonomous effects in ALS. *APC* is the most frequently mutated gene in this type of cancer and *APC* mutant cells need to outcompete wild-type intestinal stem cells within the colon crypts, resulting in the homing of the mutant cells, which drives adenoma formation [63]. *APC*-mutant tumours display a constitutive activation of the WNT pathway, and analysis of complex mouse models with an oestrogen inducible Cre expressed in villi and a heterozygous floxed *Apc* allele showed up-regulation of specific WNT-target genes, including *Notum*, which disrupts WNT binding to its receptors [63]. Studies using another tailored mouse model enabling lineage tracing of intestinal stem cells, showed significant increase in *Notum* expression in *Apc* mutant cells. Further analyses demonstrated that *Notum* is an excellent biomarker of *Apc*-mutant clones and a primary determinant of WNT signalling suppression in adjacent wild-type stem cells. As a result, Notum inhibitors are currently being developed for clinical use [63].

An abnormal immune response is of increasing interest in ALS and other neurodegenerative disease. Here again, we can take advantage of complex mouse models created in this case to address the importance of immunosurveillance of cancer cells in pancreatic and lung cancer. Determining the immunopeptidome (the peptide antigens on major histocompatibility complex class I molecules, MHC-I) in cancer was limited to in vitro investigations or studying bulk tumour lysates, neither of which provided a refined profiling over time of antigen presentation in vivo [64]. The authors addressed this question by designing mice with an inducible affinity tag (in this case a Cre-inducible exon encoding the tag) in an MHC-I gene, and then moved this allele into a well-defined mouse model of cancer containing a heterozygous *Kras* mutation and a homozygous floxed *Trp53* gene [64]. This approach showed the tumour immunopeptidome is highly dynamic, and enabled MHC-I peptides to be isolated from pancreatic and lung adenocarcinomas in vivo. From the analysis of immunopeptidomes arising from cancer cells during disease progression, the authors determined that the peptides presented were not predictable from mRNA expression analyses, but were likely driven by cryptic translation events, post-translational modifications, transposable elements and the microbiome. Interestingly, many of such phenomena are influenced by physiological cues from the in vivo microenvironment, which cannot be recapitulated in vitro [64]. The creation of this complex mouse model has given new insight into cancer-specific antigens and highlighted that antigen prediction pipelines based on mRNA abundance need reconsideration.

Although these examples tackle the biology of cancer, they clearly illustrate that we have the technology to answer difficult fundamental questions in ALS research, with specifically designed mice which enable us to study all the complexity of the in vivo situation, in each cell over lifetime, taking into account genetics, environment, age of the cell and the whole animal.

Finally, and speculatively, as in cancer it is possible sporadic ALS cases could arise from somatic mutation [65] and while evidence for this is limited, such events may in part explain the ‘hidden heritability’ of ALS [66, 67]. Somatic mutation is challenging but possible to model in vivo [68] and creation of new ‘somatic’ ALS models could provide new insight into causal mechanisms in ALS.

## Translation needs matched mouse and human trials that include biomarkers and endophenotypes

We cannot move to clinical trials without going into animal models to assess the vast array of parameters required for drug development. These include target engagement, systemic effects, formulation to assist delivery to affected tissues, pharmacodynamics/ pharmacokinetics, and dosing. Then evidence of response to treatment in a non-cell autonomous disease such as ALS is likely to be multifaceted, requiring diverse functional and biological readouts. Target engagement can be studied in cell lines, including patient-derived induced pluripotent stem cells (IPSCs), if we can assay for specific cellular hallmarks of disease, however, animal models provide a wider range of measures that can be modified by the disease and upon treatment. The choice of the most suitable preclinical strategy for drug screening is paramount and requires the most informative models of disease and pathological processes, which likely includes a combination of matching cellular and animal models.

Stage-specific therapies are essential. For example, pre-symptomatic carriers and familial ALS patients have been the subject of recent developments in modifying treatments centred on the use of ASOs to target disease-causing mutations [69]. Animal models are central to research into pre-symptomatic disease: progression from a clinically silent to manifest disease can be investigated in tailored models to assess pathology at a functional and biological level. Such models include those with conditional and inducible mutations enabling us to explore cell-specific and timing effects, as well as those expressing, for example, tags allowing us monitor the interactome in tissue- and stage-specific datasets and in response to treatments. Issues in working with mouse models, such as which genetic mutation to use, on which genetic background, at which stage of lifespan/disease development, are well known, and need to be carefully addressed, along with robust statistics on cohort sizes and sex differences.

Comparison between mouse models and patient data must be stratified by the ALS subtype under study, and with a range of prognostic, diagnostic and predictive biomarkers that can be used in both species. An area of alignment between human ALS subtypes and mouse models is the need to assess biomarkers, physiological parameters, endophenotypes and quality of life and treatment success and not ‘end-stage’ survival [70–72]. Ideally, measurable parameters reflecting improved quality of life in ALS patients, would include increased muscle function as well as reliable biomarkers monitoring altered disease progression [73]. Finally, comparison of the similarities and differences of pathology in each ALS subtype, human and in models, is essential because this may lead us to common pathways that may be tractable to shared translational approaches.

## Stratifying patients in ALS clinical trials and the need for more models to understand ALS phenotypes

Multiple clinical trials in ALS have been undertaken. A recent review established that between 2008 and 2019, 125 trials testing 76 drugs in 15,000 ALS patients were carried out [42], almost all without identifying an effective disease modifying therapy. Many factors contribute to these figures, including trial design, patient compliance and retention as well as a lack of reliable biomarkers. These challenges are now being addressed, but the phenotypic heterogeneity of ALS remains one of the biggest issues for basic and translational research, because it results in significant variability in time and site of disease onset, molecular pathologies, rates of progression and of course survival. This disease heterogeneity underlies one of the biggest single factors limiting progress in ALS clinical trials: the challenge of patient stratification [74].

The *genetic* differences between ALS subtypes are well-defined and although a few ALS genes remain to be found (25% of hereditary ALS remains of unknown cause), we already know those responsible for the majority of familial ALS. The *phenotypic* differences in ALS that may be also key to clinical stratification and trial design include rate of disease progression and possibly, site of onset; for example, ALS patients may present with involvement of bulbar innervated muscle only, for a long period or for the entire disease duration. Based on the observation that weight loss is a negative prognostic factor in ALS, a high-caloric diet was tested in a clinical trial and showed no evidence for a life-prolonging effect -- but upon *post hoc* analysis, the sub-group of fast-progressing patients had a significant survival benefit [75, 76].

Thus, it is important to factor into trial design the *a priori* classification of patient subtypes based on available prognostic models and biomarkers. As well as identifying patients likely to have a more rapid course of disease, it is critical to identify those 10–20% of ALS patients that have a survival longer than 10 years; these are patient subsets in whom treatment effect may be different in the context of a clinical trial. Randomization should also take into account age and respiratory status at entry, along with a rating of disease progression pre-entry [77]. As well as more targeted treatments for ALS subtypes, factoring in different clinical expressions of ALS may bring other benefits: for example, measurement of neurofilament concentration in blood and CSF has aided stratification in ALS and treatment response, where the goal is to slow disease progression, but it also may reduce the number of patients needed to design informative clinical trials [78]. This will be important for longitudinal studies focussing on a relatively smaller number of homogeneous patients.

With respect to the use of mouse models of ALS, a therapy developed for a specific pathology, such as an RNA binding protein dysfunction may not work in another form of ALS caused by mutation in a membrane trafficking protein, hence the need for stratification of patients with mechanistic data including from relevant mouse models. Just as importantly, while we have genetic mouse models that give phenotypic variability including speed of disease progression on different genetic backgrounds [40, 79–81] or in different environments [22], a challenge remains in that we do not yet have models for different anatomical regions of disease onset or for sporadic disease. Complex models such as cell-specific/tissue-specific inducible mutations for example, should help, but we also need the key biochemical pathways and events responsible for these human subtypes.

## The ALS drugs we may have missed

A tragic outcome of the hurdles raised by having many disease subtypes, and often low patient numbers for genetic forms of ALS, is that we may have developed drugs that work for some people but these have been lost because they failed in older clinical trials of unstratified patients relying on functional rating scores and survival as primary endpoints. Multiple compounds have been shown to be effective in *SOD1* transgenic mice, and may well have proved beneficial in *SOD1*-ALS patients; one example is the heat-shock protein co-inducer arimoclomol, which performed well in preclinical *SOD1*-ALS transgenic mouse studies [82] and was taken to a double-blind, placebo-controlled trial in 38 patients with rapidly progressing *SOD1*-ALS, 19 of whom were given placebo, 17 given arimoclomol. The study was not powered for therapeutic outcomes but showed arimoclomol was safe and well-tolerated and the treated group performed notably well in the prespecified outcome measures [83]. As the authors stated, the small sample size reflected the rarity of the *SOD1*-ALS population: estimated at 320 patients across the whole USA, not all of whom would have consented, and recruitment was in competition with two other clinical trials at the time. Although > 10% of patients with SOD1-ALS were enrolled, this was too small to go to the planned phase III trial in SOD-ALS patients [83]. Thus, the difficulties of patient stratification, including small patient numbers for most ALS subtypes, are a key issue for translation, even when well-characterised genetic mouse models are available.

## Conclusion: For successful translation we need novel, complex ALS mouse models, and we need to use their full potential

**Fig. 1:**
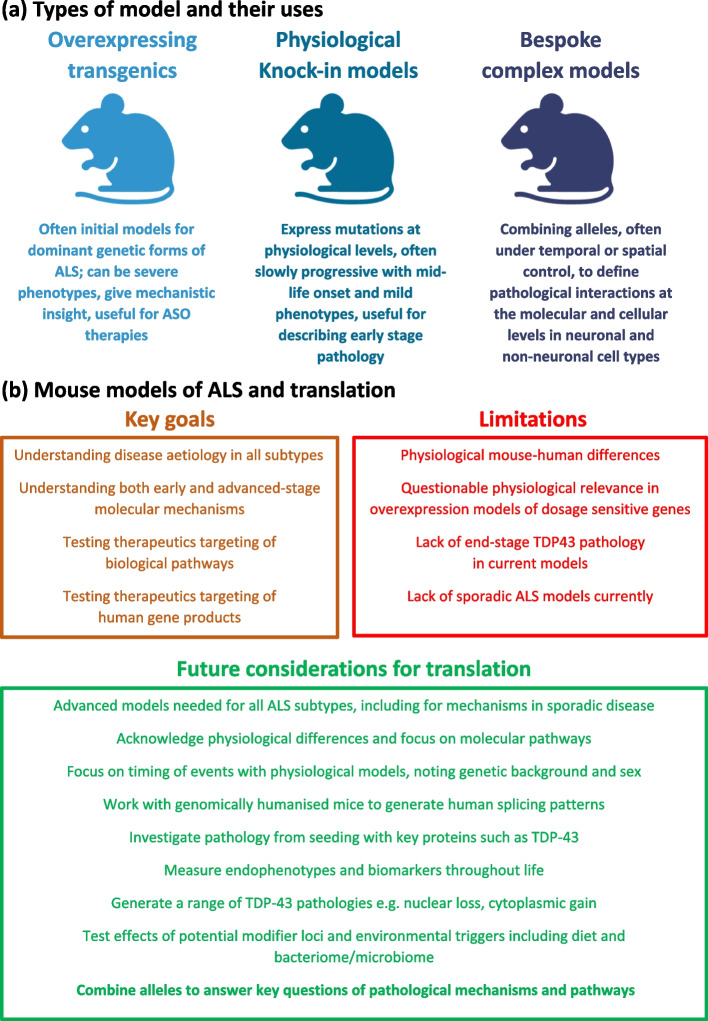
Mouse Models for ALS research

ALS research needs every model system in our armoury to dissect pathologies and develop conventional, genetic and/or cellular treatments for the many forms of this disease. For basic research studies, as we discover and validate the diverse molecular pathways that lead to ALS, tailored mouse models can be generated to determine why motor neurons die, and how we successfully stop this outcome (Table 1, Fig. 1). For example, tissue specific and cell autonomous effects can be investigated by conditional and inducible strains in which a mutation can be switched on, or off, at specific times, in specific tissues. Such experiments depend on a range of drivers of recombinases (such as Cre- or Flp-) and appropriately engineered mouse loci, including those with exons duplicated and flanked by LoxP or FRT sites to switch wild-type and mutant sequences at will in vivo (for example, [84, 85]). Pathological processes within individual cell types can be investigated and validated by optogenetic and other approaches using genetically encoded sensors [86] and reporter strains, for example, to assess effects in upper motor neurons [87] or the effects of different transcription factors [88] or alterations of neuronal proteostasis [89].

**Table 1:** Questions for translation from ALS mouse models to the clinic

• What are the disparate primary causes and pathogenic processes that lead to the similar clinical and cellular outcomes of sporadic and familial subtypes of ALS?
• Can we translate treatments for heterogeneous forms of ALS, without knowing specific molecular mechanisms?
• Can we create models for sporadic ALS?
• Have we missed drugs that would have been successful in people with *SOD1*-ALS?
• Are any treatments developed in SOD1 transgenics, other than ASOs for SOD1 itself, likely to work in the 98% of patients who do not have *SOD1*-ALS?
• How do we resolve the tension between the biology/pathology of ALS models versus the constraints of time/financial realities in pharma/biotech/academia?
• Can we create complex mouse models that give us disease progression/biomarkers/pathologies that are helpful for translation for each ALS subtype? How are these paid for?
• What markers of disease progression (biomarkers/endophenotypes), not of endstage, should we use in mouse preclinical studies to give us granular longitudinal data on drug effects prior to translation into humans?
• What markers of disease progression (biomarkers/endophenotypes) should we use in human clinical trials?
• How do we overcome the difficulties of patient heterogeneity and small numbers for each subtype, and trial stratification in translation from genetic mouse models to human ALS patients with sporadic and familial disease?

Genetic interactions can be explored through multiple hit models -- different engineered alleles bred together, to dissect mechanisms of disease onset and progression [90]. Individual alleles may manifest no, or mild phenotypes, but together can provide powerful insights for translation; a recent example from cancer research comes from mice created by cross-breeding 15 different alleles each involved in diverse phenomena such as apoptosis, the cell cycle, development, the immune system – these mice are providing new insights, and ultimately new treatments, for multiple myeloma, and importantly at different clinically relevant stages of disease [91].

‘Hits’ can also be environmental factors, in order to tease out gene x environment interactions: for example, a recent study of low dose of the toxin β-N-methylamino-L-alanine, which is associated with Guam-ALS, given to mice expressing a TDP-43 transgene with a mild phenotype, showed a worsening of motor phenotypes in the treated versus untreated transgenic mice [92].

Models expressing endogenous levels of human ALS genes offer the potential of maximum physiological relevance but may be hampered by the short lifespan of the mouse which limits disease progression and robust readouts for translation. Here, it is possible that complex approaches including seeding key proteins may provide new avenues of research. In studying prion diseases, Alzheimer’s disease, tauopathies, and alpha-synuclein pathology, transmission of patient-derived proteopathic seeds into humanised mice accelerates pathological cascades and produces relevant strong phenotypes and/or pathology [93–98]. This may be an important avenue of investigation in physiological ALS models.

For translational studies, mice play a particularly important role because drug development requires testing in animal models, and for largely pragmatic reasons of ability to engineer their genomes, and cost, these are the chosen species for preclinical trials. The need for mouse models is clear even when the therapy is not targeted to a particular pathological process: ASO approaches for *SOD1*-ALS and *FUS*-ALS have gone forward to the clinic after testing in mice and giving safe, positive outcomes, although the key pathological processes resulting in motor neuron death in vivo are not clear [10, 99, 100].

In this age of personalised medicine, there are important lessons from the success of cancer researchers who are similarly dealing with disparate diseases, often of unknown causes, affecting multiple pathways, sometimes with very small numbers of patients. Cancer studies use tailored mouse models and tailored drugs in screens of highly profiled patients [101–104]. ALS researchers have similar difficulties such as the small size of patient cohorts and our current lack of key pathological pathways. A major additional problem of ALS and other neurodegenerative conditions is that we cannot sample and examine the affected tissue prior to and following enrolment in trials [74]. Nevertheless, in-depth studies of endophenotypes and biomarkers, and international cooperation for stratified patient trials and multi-centre engagement is advancing progress in the clinic [72, 74, 105, 106]. Now we need a wide array of mouse models tailored to address specific questions to further our understanding of pathomechanism and intervention in all ALS subtypes.

## Data Availability

Not applicable

## Abbreviations

ALS: Amyotrophic lateral sclerosis
*APC*: Adenomatous polyposis coli (gene)
ASO: Antisense oligonucleotide
CST: Corticospinal tract
EGFP: Enhanced green fluorescent protein
FUS: Fused in sarcoma
HNRPNA1: Heterogenous nuclear ribonuclearprotein A1
IPSC: RV longitudinal strain
Kras: Kirsten rat sarcoma virus gene
LMN: Lower motor neuron
MATR3: Matrin 3
MHC I: Major histocompatibility complex class I molecules
OLE: Open-label extension
OPTN: Optineurin
NFH: Neurofilament heavy chain
*TARDBP*: Gene encoding TAR DNA Binding Protein 43
TBK1: TANK-binding kinase 1
TDP-43: DNA Binding Protein 43 (protein)
*TRP53*: Gene encoding the p53 tumour suppressor
SETX: Senataxin
SOD1: Superoxide dismutase 1
SQSTM1: Sequestosome 1
STMN2: Stathmin 2 gene
UNC13A: Unc13 homolog A
UMN: Upper motor neuron
VCP: Valosin-containing protein
WNT: Wingless-type protein
